# The Need for Machines for the Nondestructive Quality Assessment of Potatoes with the Use of Artificial Intelligence Methods and Imaging Techniques

**DOI:** 10.3390/s23041787

**Published:** 2023-02-05

**Authors:** Marek Danielak, Krzysztof Przybył, Krzysztof Koszela

**Affiliations:** 1 Department of Biosystems Engineering, Poznań University of Life Sciences, Wojska Polskiego 50, 60-625 Poznan, Poland; 2 Lukasiewicz Research Network—Poznań Institute of Technology, Starołecka 31, 60-963 Poznan, Poland; 3 Department of Dairy and Process Engineering, Faculty of Food Science and Nutrition, Poznań University of Life Sciences, Wojska Polskiego 31, 60-624 Poznan, Poland

**Keywords:** potato, tuber, quality assessment, sorting machines, grading, ultrasound, neural networks, machine vision, nondestructive method, noninvasive method

## Abstract

This article describes chemical and physical parameters, including their role in the storage, trade, and processing of potatoes, as well as their nutritional properties and health benefits resulting from their consumption. An analysis of the share of losses occurring during the production process is presented. The methods and applications used in recent years to estimate the physical and chemical parameters of potatoes during their storage and processing, which determine the quality of potatoes, are presented. The potential of the technologies used to classify the quality of potatoes, mechanical and ultrasonic, and image processing and analysis using vision systems, as well as their use in applications with artificial intelligence, are discussed.

## 1. Introduction

Potatoes are one of the most important crops in the world. Over the last two decades, i.e., from 2000–2020, an 11% increase in their production in the world can be observed, from approximately 322 million tons of potatoes produced in 2000, to approximately 354 million tons in 2020. Asia had the largest share of potato production: 49.7%, Europe: 30%, and North and South America: 12.5%. In 2020, the world’s largest potato producers were China (approximately 78.2 million tons), India (approximately 51.3 million tons), and Ukraine (approximately 20.8 million tons). Their total share exceeded 40% of the total world production of potatoes. In 2020, the world’s potato cultivation area decreased by about 17% compared to 2000, from approximately 19.9 million ha in 2000 to approximately 16.4 million ha in 2020; nevertheless, a 34% increase in potato productivity in the world was observed, from approximately 16.2 tons/ha in 2000 to approximately 21.8 tons/ha in 2020 [1]. In the commodity structure of agricultural production in 2020, the potato, similarly to palm oil, accounted for 4% of world crop production in 2020, just behind wheat, corn, rice, and soybeans [2,3].

In Poland, for many years there has been a clear downward trend in the use of potato acreage, a slight decrease in their harvest, and an increase in yields [4,5]. In 2021, the potato cultivation area decreased by approximately 38% compared to 2010, while the use of potato acreage increased by approximately 4% compared to 2020. Potato harvest in 2021 was lower by 11% compared to 2010 and lower by 10% compared to 2020. In 2021, the yields obtained from 1 ha were approximately 44% higher compared to the yields obtained in 2010 and were approximately 14% lower compared to 2020. In 2020, compared to 2010, Poland dropped from seventh to tenth place in the world in potato production. Thus, its share in the world decreased from 2.6% recorded for 2010 to 1.8% in 2019 [6]. In the EU potato production in 2020, compared to 2010, Poland dropped from first to third position and its share decreased from 31.4% in 2010 to 14.5% in 2020 [6]. In 2020, compared to 2010, potato exports decreased by 31%, but were higher by approximately 2.4% compared to 2019 [6]. Among the reasons for the decrease in potato production in Poland are the constantly decreasing consumption of potatoes, the replacement of potatoes in the fodder and distillery industry with more profitable crops, fluctuating sales prices, an increase in the turnover prices of means of production, difficulties with selling crops by small producers and difficulties in exporting resulting from strict requirements in terms of commercial quality related to quarantine bacterial diseases [7]. In 2020, there was a decrease in the consumption of potatoes per capita by 15% compared to 2010, and, at the same time, an increase in consumption by 1% compared to 2019 [6]. Over the years 2010–2019, a 3% decrease in potato consumption in the world was observed, from 33.36 kg/year in 2010 to 32.41 kg/year in 2019. The largest increase in potato consumption was recorded in Asia, by 7%, from 28.14 kg/year in 2010 to 30.28 kg/year in 2019. In North and South America, a slight increase of about 1% in consumption was observed, from 35.72 kg/year in 2010 to 36.01 kg/year in 2019. In Europe, Asia, and Oceania, a decrease in potato consumption was recorded, which was, respectively, in Europe by 10%, from 79.56 kg/year in 2010 to 71.23 kg/year in 2019, Africa by 20%, from 18.17 kg/year in 2010 to 14.4 kg/year in 2019, and Oceania by 20%, from 39.36 kg/year in 2010 to 34.63 kg/year in 2019. In the European Union, the average consumption decreased by 5% from 64.75 kg/year in 2010 to 61.25 kg/year in 2019. In Poland, a decrease in consumption was observed by 12% from 113.32 kg/year in 2010 to 99.42 kg/year in 2019 [1].

According to the suggestions of Sosnowska, in Poland it is estimated that approximately 90% of the potato harvest is intended for storage annually. Depending on their intended use, the time and amounts may vary to 6–7 months for seed potatoes, 1–9 months for ware potatoes, and 10 months for potatoes intended for processing. Due to a long period of potato storage, it is extremely important to provide the tubers with the most favorable conditions, which will translate into good quality and minimize losses [8]. Loss of all food produced is inevitable due to naturally occurring physiological processes such as respiration, transpiration, germination, and disease [8]. It is estimated that the potato yield can be attacked at any phase of harvest development by nearly 160 diseases caused by fungi, bacteria, viral infections, nonparasitic causes, or due to obscure causes [9]. Undetected potato diseases lead to the spread of pathogens of infected potatoes in warehouses, which generates greater storage losses, and on plantations—in the case of infected potatoes intended for seed material, which translates into lower yields and their quality. Wastes occur at every stage in the product supply chain. They are between 20% and 30% [10,11,12]. According to Sosnowska, in Poland potato losses and losses in the total harvest balance usually range from 5–30%, sometimes as much as 80% [8]. Losses at the stage of harvesting and transport in the absence of appropriate qualifications and facilities for harvesting machines and undeveloped road infrastructure may amount to 12.45 and 11.7%, respectively, as showed by research in Ethiopia [13]. Tests in Austria and Germany showed that potato losses during harvest can range from 1–9% [14]. Damage caused during harvesting contributes to greater losses of vitamin C and water, and loss of firmness [13]. In the UK, based on data collected between 2004 and 2008, potato losses in individual links of the food supply chain amounted to, at the stage of sorting, from 3–13%, at the stage of storage from 3–5%, packaging from 20–25%, and sale 1.5–3%. The main causes of losses at the sorting stage resulted from external damage to the potato and its germination. Losses during storage and packaging resulted mainly from inappropriate temperature and inaccuracy of forecasting the demand for potatoes. Packing losses resulted from storage damage [10]. Total losses in the world of potatoes from 2010 to 2019 were stable and amounted to 9–10%. In 2019, the biggest losses were in Africa 15%, Asia 10.5%, America 8.8%, and Europe 8.7% [1]. In Poland, losses in stored potatoes over the last 10 years from 2011–2022 are stable and amount to about 11% [15]. In the 2021/2022 season, approximately 4 million tons (56% of the 2021 harvest) were allocated for the storage of potatoes. The largest losses were recorded for the Podkarpackie Voivodeship (19% and Łódzkie), while the smallest for the Mazowieckie Voivodship (approximately 8.9% and Kujawsko-Pomorskie approximately 8%) [15,16].

Reducing losses is extremely important in ensuring the necessary amount of food for people around the world. The world population in 2021 increased by more than 1% compared to 2020 and amounted to 7.87 trillion [17]. As the population grows, so does the need for food. According to the World Food Program agency, in 2030, the number of people severely affected by hunger has increased, from 619 million people in 2019, by 31% to 811 million in 2020 [18]. It is estimated that world food demand will increase from 35% to 56% between 2010 and 2050. According to forecasts, in these years, the number of people severely affected by hunger will fall by 91% to 8% [19]. Increasing agricultural productivity and sustainable food production, increasing the nutritional value of agricultural products, and obtaining resistance to biotic and abiotic stress will increase food security in the world [18], ensuring reduced food losses and waste, as well as increasing consumer requirements resulting from the growing competition on the potato market. This will result in the need to supply the market with agricultural products of the highest quality in order to guarantee that the appropriate quality standards of the product are met for the customer and the consumer; thus, sorting is carried out. Sorting begins in the field, where the harvesters preseparate the potatoes from the haulm and stone soil. The further sorting out process takes place in the storage rooms. The selection of potatoes in these facilities is carried out in two stages: before loading into the storage chambers, where impurities in the form of stones, haulm, and soil are separated, prefractionated into seed potatoes, ware potatoes, and for food processing, and in the second stage before the potato is sold, to accurately calibrate the tubers, clean the ware potato, and remove defective material [20]. However, despite the fact that the quality control of agricultural produce is supervised by trained inspectors employed on the sorting line, who manually sort the vegetables, assess their quality and make selections, rejecting all those that are undesirable in the further processing process, it often happens that due to the large volume production for inspection, inadequate training, insufficient experience, or human eye error, a product that does not meet the appropriate standards is sold: it is of inferior quality, unfit for consumption, damaged, or infected. In order to eliminate the human factor and thus reduce sorting errors, research has been conducted for many years on the development of automatic sorting machines. The developed technologies enable verification of agricultural products more precisely and reliably than the human eye. They increase sorting efficiency and ensure high repeatability of classified products.

The quality of potato tubers is not only important for the food and processing industry in the form of French fries, crisps, or dried potatoes. Starch is used as a raw material for the production of fat-free meat products or biobased plastics that are used in the production of containers for packaging, mulch films, and trash bags. The plastics based on starch are characterized by high transparency. Potatoes are also used in the pharmaceutical industry for the production of drugs and bioactive compounds for the human digestive tract in order to increase its effectiveness [21]. Potatoes are used also for making vodka or other fermented drinks that are indicated for people who are allergic to dairy products and are vegetarians [22].

## 2. Qualitative Assessment of Potatoes in Terms of Taking into Account the Nutritional Value

Potato is a product that is well known and utilized worldwide. Thanks to its rich nutritional properties, it is eagerly consumed all over the world. It can be served in various ways: boiled, fried, dried, or as an ingredient in ready-made meals. Due to this, special attention should be paid to its production to ensure adequate preharvest and post-harvest quality during storage. This entails assessing both its internal and external quality characteristics to ensure continuity of supply to consumers and the processing industry [23,24]. The qualitative features consist of many closely related and genetically controlled parameters, the share of which depends on appropriate agrotechnical and post-harvest treatments, climate, and specific terrain conditions [23]. Processed potato products are usually perceived as negative nutritional attributes as they are high in fat and sodium and/or as a significant source of carbohydrates in the form of starch. However, potatoes also have positive attributes which are not as well known, such as phytochemical content, micronutrient retention, and starch resistance [25]. Potatoes are a wholesome, nutritious, and nonfatty vegetable that contains many valuable nutrients for a sustainable diet [26]. Benson suggests that consuming one pound of potatoes can satisfy the hunger of many people whilst only providing 440 calories [27]. Potatoes have approximately 78% water, 22% dry matter (specific weight), and <1% fat. Dry matter consists of approximately 82% carbohydrates: mainly starch, which consists of amylopectin and amylose in a constant ratio of 3:1, and dietary fiber and a small amount of sugars [27,28,29]. Starch is easily digested only after cooking, resulting in a higher glycemic index, and only a small amount of residue remains undigested due to disruption in granular and cell wall structure, but when it is raw it is nearly indigestible, as starch has gelatinized [21,29]. Another valuable nutrient found in potatoes is protein. Even though they contain small amounts of it (approximately 0.6–12%), their nutritional value is still characterized as higher than that of cereals and soybeans. Potatoes also contain many vitamins and minerals, such as vitamin C, iron, thiamine, and folic acid [26]. A medium-sized baked white potato with skin weighing 148 g is a source of vitamin C (24% DV), vitamin B6 (23% DV), potassium (23% DV), dietary fiber (13% DV), and magnesium (10% DV) on a 2000 kcal/day diet [30]. There is a direct proportional relationship between the physicochemical and functional properties of raw potato tubers and the quality of finished products [31]. Texture, appearance, taste, and nutritional value are the four main factors of food quality [32]. The main quality parameters of potatoes intended for cooking are consistency, color, taste, and smell. Value dry matter and starch content are critical to the textural and rheological properties of cooked as well as raw potatoes [33]. Sedaghi et al. concluded that crispness and crunchiness are strong indicators of desirable French fry texture attributes [34].

The potato texture is correlated with the dry matter content [26]. Starch is closely correlated with the content of dry matter; both are among the most important technological features of tubers for direct consumption, for food and nonfood processing. Zarzecka found that the cultivars and weather conditions significantly affect the content and yields of dry matter and starch. Lastly, the herbicides and biostimulants determinate the starch accumulation as well as dry matter and starch yields [35].

Flour and dry potatoes have a high content of dry matter, while wet potatoes have a low content. Potatoes should have their own specific potato flavor and aroma without any foreign smells. When eaten in large quantities, some substances in potatoes are unhealthy, for example, acrylamides (which are formed during food processing such as frying potatoes to make French fries), glycoalkaloids, and nitrates [36]. Glycoalkaloids can be toxic if consumed in high quantities, although dozens of recent studies show potential health-promoting effects of glycoalkaloids, especially anticancer activity in animal and cell studies [37,38,39]. Glycoalkaloids are important components for determining the taste (TGAs), such as solanine and chaconia, which are present in all potatoes in small amounts (2–15 mg/100 g of fresh tuber weight). Tubers with higher amounts of >20 mg/100 g have a bitter taste and are potentially toxic. A greenish surface of the tuber often indicates the presence of mustard, although its development is independent of the green color. Research based in Poland shows that sunlight significantly contributes to the growth of nitrates and glycoalkaloids in potato tubers, the action of which causes the production of chlorophyll and the beginning of chemical reactions in the outer layer of the tuber. After two weeks of time of light exposure, the increase of TGA ranged from 20.67 mg∙kg^−1^ for variety Vinieta to 54.67 mg∙kg^−1^ for variety Irga, while the increase in nitrates ranged from 11.67 mg∙kg^−1^ for variety Bellarosa to 27.50 mg∙kg^−1^ for variety Irga [40]. The most important qualitative characteristics of potatoes intended as a raw material for food processing into chips are dry matter, specific gravity, reducing sugars, and color [41]. In the case of potatoes intended for fries, the most important parameters include dry matter, low level of reducing sugars, taste, and the presence of defects. Dry matter is measured as tuber specific gravity and is negatively correlated with water content. During the frying process, there is a loss of water from the potatoes, as a result of which the weight of the raw material decreases, which translates into lower profits. The specific gravity of the tuber also directly affects oil absorption during frying. The lower the specific gravity, the higher the oil absorption, which also results in higher production costs. The color of the fries depends on the content of reducing sugars, which react with amino acids during frying as a result of nonenzymatic browning reactions, creating a dark color. The sugar content, which depends on the variety, agrotechnical conditions, tuber maturity, and storage conditions, should therefore be as low as possible. Scientists have proven that the most important soil nutrient factors affecting the quality of tubers is pH value, which affects the content of potato starch, reducing sugars, soluble protein, and soil nitrate nitrogen, which in turn affects the content of soluble sugars, the intensity of browning, polyphenolic activity in tubers, and the content of vitamin C [42].

The color of the fries should be uniform and free of any defects resulting from sunburn, reticular necrosis, sugar ends, carbon spots, bruises, or light or dark brown areas [26]. The Kulfri Fryson variety, which has a high content of pulp (23.35%) and starch (28.52%) and the lowest chips color score of 1.5 (on a scale of 1–10) [41] has been recommended for the production of chips in India [41]. Potatoes have many health benefits. The antioxidants they contain include ascorbic acid, carotenoids, lutein, zeaxanthin, and violaxanthin, which improve oxidative stress. The compounds present in them, such as phenolic acids, carotenoids, and anthocyanins, have a positive effect on the lipid metabolism and can reduce inflammation in the body [43,44]. The phenolic compounds present in the potato skin, chlorogenic acid and anthocyanins, together with antioxidants, fight cancer cells and have a pro-health effect on the heart and vascular system by minimizing the risk of hypertension. This is because the potassium content in potatoes is one of the most important sources among vegetables and fruits that also plays an important role in bone health [30,45]. Research suggests that potato nutrients and composition may have favorable impacts on gut bacteria, playing a critical role in the metabolism of potato bioactive components [44]. Resistant starch RS2 and RS3 are contained in raw and cooked or cooled potatoes, helping to reduce the risk for insulin and leptin resistance and playing a role in satiety [28,44,46]. Potatoes are included in the list of food-based dietary guidelines of countries that recommend eating potatoes due to their health properties: Portugal recommends 1 and 1/2 potato (medium size = 125 g) per meal, Italy recommends servings potatoes/week (1 serving = 200 g) [47].

There are about 4000 native species in South America [27], and at the same time the physical and chemical parameters of each of them are different, which is why it is so important for the technological process to describe the properties and nutritional values of each variety for the benefit of the consumer.

In Poland, detailed legal standards for the marketable quality of potatoes are contained in the Regulation of Agriculture and Rural Development of 29 October 2003 on detailed requirements for the marketable quality of potatoes, and its subsequent amendment in the Journal of Laws. U. 2019 item 139. According to the applicable regulations, potato tubers should be of appropriate size. The minimum transverse diameter of round and round–oval early (baby) potatoes should be 28 mm. Smaller vegetables are allowed if their mass share does not exceed 3% and their diameter is at least 15 mm. In the case of edible potatoes, the minimum transverse diameter of round and round–oval tubers is 35 mm, for elongated tubers, the transverse diameter is 30 mm, while the longitudinal diameter should be twice as large as the latitudinal diameter. Vegetables with a smaller size are allowed for marketing; the mass share of tubers with a smaller diameter may not exceed 2%, however, their diameter may not be less than 28 mm. The maximum mass share of potato tubers that are green on at least 1/8 of the surface in the case of early potatoes should not exceed 1%. On the other hand, in the case of edible potatoes, the total share of green tubers with internal defects in the form of rust, black, gray spots, or hollow hearts should not exceed 2%. The maximum mass fraction of early and edible potatoes affected by gangrene, dry or wet rot, and late blight is 1%. In the case of mineral impurities (lumps of earth or stones) or in the form of organic impurities (parts of straw, haulms, weeds, or broken sprouts), the mass fraction should not exceed 2% for early potatoes and 1% for ware potatoes, respectively. The weight share of ware potatoes affected by common scab, with symptoms of infection in the form of spots covering more than ¼ of the surface of the tuber, should not exceed 3%. In the case of damage caused by mechanical harm or pests, for early potatoes, it is visible at a depth of more than 3.5 mm, whereas in tubers with shallower surface damage, it is of at least 1/5. In both cases, the mass fraction should not exceed 2%. For ware potatoes with mechanical or pests damage, occurring at a depth of more than 5 mm, the mass fraction should not exceed 3%. In the case of ware potatoes, the mass fraction for tubers clearly different from the varietal type, deformed, baby-shaped, or spindle-shaped tubers, the mass fraction must not exceed 3%. The percentage of ware potatoes whose tubers are immature, flaky, with an undeveloped, uncorked skin that flakes when touched, should not exceed 2%. The weight percentage of tubers of other varieties for both ware and early potatoes should not exceed 2%. The maximum total weight share of early potato tubers, including the shares of tubers that are green, affected by rot, with mineral or organic impurities, and undersized, should not be greater than 5%. In the case of ware potatoes, the maximum proportion by weight of tubers, consisting of the maximum proportions of tubers: green, with internal defects, affected by rot, with mineral or organic impurities, undersized, or damaged, is 6% and 8% for the criterion including the above proportions together with proportions of misshapen, immature, or other varieties. Including, as in the case of early potatoes, the share of green tubers, affected by rot, with mineral or organic impurities, the share of tubers with a diameter smaller than the permissible one, plus the share of tubers with internal defects, caused by scab and damaged tubers, should not exceed 5%. Edible potatoes allowed for trade should not be sprouted more than 3 mm. It is unacceptable to introduce to the market potatoes that are excessively moist on the surface, boiled, whose tubers are devoid of turgot, have a clear darkening of the flesh between the surface of the tuber and the ring of conductive bundles, or whose flesh is cracked. Bulbs with some or all moldy surfaces are unacceptable. Frozen tubers, soft tubers that ooze water, or have a darkened zone or an irregular gap between healthy and dead tissue are not suitable for marketing. Potatoes contaminated with plant agents, solid or liquid residues, as well as tubers with foreign odors, are not suitable. The provisions of the regulation apply only to potatoes intended for commercial purposes; they do not apply to early and ware potatoes intended for processing. The proper application of the above provisions is aimed at ensuring food safety, improving the quality of potatoes, which not only affects the health and satisfaction of consumers, but also has a significant impact on the development of an economy based on the principle of fair competition [48].

## 3. Application of Mechanical Methods in Machines for Nondestructive Quality Assessment of Potatoes

Manufacturers of sorting machines are constantly outperforming each other in designing and introducing more innovative solutions to the market that guarantee high quality of sorting. The main parameters for sorting potatoes are thickness, width, and weight of the tuber [49]. On the Polish market, mechanical sorters for classification in terms of size, length, width, or thickness are commonly used, such as sieve sorters mounted on rockers with reciprocating motion, circulating mesh, circulating roller, circulating roller, and vibrating length. Drum sorters are used less frequently due to causing damage [49].

### 3.1. The Length Vibrating Sorter

The length vibrating sorter consists of a frame, a set of sorting plates, and outfeed conveyors. The sorting plates are supported on vibration isolators on the frame. They are excited to vibrate by means of an electric vibrator and fixed at a slight angle to the level with an inclination, thus enabling the gravitational movement of the raw material towards the chute. The plates are made of profiled sheets with narrow ribs in the shape of grooves, set transversely to the direction of movement of the raw material, thus ensuring the appropriate orientation of vegetables, the longer part of which falling into the depressions is set parallel to the direction of the sorter’s operation. Their spacing is adjustable and allows the rejection of vegetables that are too short on discharge conveyors suspended under the plates, in the places of the adjusted slots. The device enables the selection of raw material in terms of length into one to three fractions and oversort.

### 3.2. The Drum Sorter

The drum sorter consists of a frame, drum, and chutes. On the frame, at a slight angle inclined to the horizontal, the drum shaft is mounted with three hoops, around which the net with different mesh sizes is wrapped. In the upper part of the drum there is a chute for the drum, while there are chutes under the drum. The drum is driven by an electric motor with a belt transmission. The drum sorter is used to sort potatoes in terms of size into seed potatoes and ware potatoes, and to remove impurities smaller than the smallest mesh of the sorting net. They are designed for sorting into three fractions: two sorts and supersort. Drum sorters are no longer produced; however, used sorters can still be purchased at online auctions or found on small farms [50].

### 3.3. The Sieve Sorter

The sieve sorter consists of a frame, a set of sorting sieves, a charging hopper, bagging machines, and a drive system. Optionally, it can be equipped with an inclined feeding conveyor or a sorting table. Sorting sieves are placed on the frame at a slight angle of 3–5° [49] and set in reciprocating motion by using an electric motor with a belt transmission and a connecting rod mechanism [50] or by means of shakers or vibration exciters [50]. The sieves are arranged in layers, one above the other, arranged according to the mesh size, from the sieves with the meshes of the largest dimeter placed at the very top of the sorter to the sieves with the meshes of the smallest diameter arranged at the very bottom of the device. Each sieve has a separate chute. Under the influence of the reciprocating motion, the potatoes move along the upper sieve towards the chute. Tubers with a diameter smaller than the mesh size of the sieve fly over the sieve placed below and stop on the sieve whose mesh size is larger than the diameter of the tuber. Sorters enable fractionation into 3–5 groups [49].

### 3.4. The Mesh Circulating Sorter

The mesh circulating sorter is made of a frame on which rollers are mounted at a slight angle to the horizontal, with a sorting belt wrapped around them, which has cut out eyes and is usually made of flexible material that protects the tubers from damage during sorting. The device is equipped with two drive units. The first one, responsible for moving the belt, consists of an electric motor and a gear coupled with the belt drive shaft. The second drive, responsible for tamping the tape, includes an electric motor, gears, and an eccentric mechanism. Stepless adjustment of the belt advance or its boosting is provided by a variator or an inverter. Under the sorting belt, there is a conveyor carrying the sorted raw material. The device sorts into one caliber and oversort. In order to obtain a larger amount of fractions, it is necessary to place several circulating sorters in a line in the amount depending on the user’s preferences. The sorter can work independently or cooperate with a feeding conveyor or a selection table. The mesh circulation sorter is characterized by high efficiency and lower forces acting on the tubers during sorting [51].

### 3.5. The Roller Sorter

The roller sorter consists of a frame, a set of sorting rollers, a drive system, a chute, and discharge conveyors. Optionally, the device can be equipped with an inclined collecting conveyor. The rollers are made of stainless steel. They are arranged at a slight angle to the horizontal, perpendicular to the sorter’s frame and connected with each other by a chain. The chain is driven by an electric motor via a gearbox. The chain feed speed is regulated by an inverter or variator. The roller spacing is mechanically adjustable. The discharge conveyors are mounted under the rollers. Sorting is carried out by means of rotating and moving rollers, which gradually change their spacing during the chain movement and thus allow the tubers to fall onto the discharge conveyors [51].

### 3.6. The Spiral Sorter

The spiral sorter is built with a frame, sets of spiral rollers, outfeed conveyors, drive unit, and chute. The rollers are mounted on the frame, transversely to the machine. They are placed at various intervals, gradually increasing until the end of the frame. Their spacing is mechanically adjusted. Adjacent rollers rotate in opposite directions. The drive to them is transmitted through the engine and transmission. Each of the rollers usually has a separate drive. Under the rollers, in the slots, there are conveyors that transport the sorted raw material [51].

An advanced size sorting machine for potatoes has been developed, which is equipped with a sorting surface that is made up of infinitely closed elastic elements (belts) wrapped around pulleys. Thanks to the difference in the speed of the belts, the potato moves on the sorting surface and adapts to the openings between the belts, falling into the discharge conveyors fixed under the sorting surface. The selection of the optimal operating parameters of the machine is as follows: the speed of a slow-moving belt is 0.4 m/s, the speed of a fast-moving belt is 0.6 m/s, and the angle between adjacent belts is 0.46′26, allowing for obtaining 0.95% accuracy of sorting potatoes in terms of size and negligible, not more than 0.5%, mechanical damage to tubers caused during sorting. The machine for every 10 tons sorted allows you to reduce production costs by Uzb 900,000–1,000,000 [52].

## 4. Application of Ultrasound Methods

For many years, work has been carried out on the use of ultrasound in the food industry for quality testing of food products and its modification due to its low cost, simplicity, and energy efficiency [53,54,55,56,57,58]. The ultrasound method is successfully used in two bands: in the band from 100 kHz and intensity below 1 W·cm^2^ (loop-powered ultrasound (LPU)) to monitor the composition and physicochemical properties of agricultural produce during its processing or storage, and in the band from 20–500 kHz and intensity 1 W·cm^−2^ (high-powered ultrasound (HPU)) to study changes in the mechanical, physical, and chemical/biochemical properties of crops during many food processing operations, such as extraction, freezing, drying, emulsification, and inactivation of pathogenic bacteria on surfaces in contact with food. The speed of ultrasonic wave propagation in the tested material is very sensitive to the molecular organization and intermolecular interactions, which allows for the determination of the composition, structure, physical state, and various molecular processes such as the detection of defects and foreign bodies, phase transitions, and crystallization. Other parameters that correlate with the parameters of the materials are the attenuation coefficient and acoustic impedance. Two methods are used: the pulse technique and the “through transmission” technique. In the first method, one transducer is used, which generates and receives a sound impulse that passes through the structure, is affected by changes in the structure, and, reaching the outer wall, returns to the recorder. Based on the measurement of the time between the emitted sound signal and the echo, the ultrasonic speed and attenuation factor are calculated. In the second method, two transducers are used: a pulse generator and a detector mounted on both sides of the sample. The wave passes through the sample and is received by the second transducer. Based on two signals, the time of passage of the wave is measured and the speed is calculated. In both methods, ultrasound is generated using appropriate transducers by changing the electric current [59].

Scientists have demonstrated the feasibility of using ultrasound to detect hollow heart disease in potatoes using ultrasonic sensors in an aquatic environment. The resonance of crystal sensors was observed at a frequency of 175 Hz [60].

Scientists investigated the possibility of using a pulse transmission method using longitudinal ultrasonic waves in the range of 50 kHz to 1 MHz for disease classification during tests in the aquatic environment. The average ultrasonic velocity at 100 Hz was 824 $\frac{m}{s}$ during measurements. It has been observed that the disease can be easily detected by adjusting the attenuation coefficient for healthy and diseased potatoes. The attenuation coefficient of defective potatoes was much higher than for healthy potatoes. Setting the threshold value of the attenuation coefficient of 47 dB for the prediction model created on the basis of the attenuation coefficient values for the frequency of 100 Hz allowed to achieve the accuracy of identifying the disease hollow heart at the level of 92% [61].

Researchers investigated the possibility of using a transmission method with ultrasonic waves for a frequency of 250 kHz in a dry environment for identifying hollow heart in tubers. Based on the time domain analysis, it was observed that the duration of the signal is much longer and more jagged than for a healthy potato. Based on the analysis in the frequency domain, it was noticed that the amplitude of the damaged potato signal spectrum is much smaller, and its character is more jagged due to reflections and interference occurring inside the tuber than for healthy potatoes. Power spectral density analysis showed a similar, lower amplitude of power spectral density for hollow heart potatoes. It has been observed that the threshold value of the power spectral density less than 0.2 v^2^ Hz could provide an effective method for the identification of quantitative evaluation of hollow hearts [62,63].

Jivanuwong conducted research on the use of dry-coupled ultrasound with frequency of 50 kHz and a power level of 0.22 W for identification of hollow heart in the transverse direction along the longitudinal axis of the tuber. Transmission losses for hollow heart potatoes have been observed to be greater than 0.28 dB/mm. Prediction based on this threshold value allowed the estimation with an accuracy of 98%; however, its ability to predict hollow hearts in potatoes was limited to the minimum size of the cavity, 0.5 cm. It has been observed that physical parameters such as tuber length, diameter, shell weight, and modulus of elasticity have a weak correlation with ultrasonic parameters such as ultrasonic velocity, root mean square voltage of transmitted signal, or attenuation coefficient [64].

Research was conducted on the use of a noninvasive and noncontact ultrasonic method in an aqueous environment to assess the quality of samples, and the possibility of using ultrasonic probes in the development of an underwater sorting machine was analyzed. The LabVIEW software was used to acquire measurement data from the ultrasonic sensor, and calculate the spectral moment and function for potato classification. A clear dependence of the size of the power spectral density on the density of potatoes was observed, which gives rise to the possibility of using the power spectral moment, calculated from the integral of the power spectral density, for potato density sorting applications. The power spectral moment for evaluated potatoes with no hole, one hole, and two holes were, accordingly, 4210, 2039, and 1275 Hz. It was observed that the threshold value of power spectrum moment of 75 v^2^ Hz could be used for sorting potatoes with different density [65].

The researchers proved the possibility of using an acoustic sorting system to detect a hollow heart in a potato. Three features extracted from the microphone signal were used to build the predictive model: integrated absolute value of microphone output signal, and the other two for analysis were number of data points in the digitized microphone signal following impact that have slopes, and magnitudes below preset threshold levels. The analysis showed that the impact location had little effect on the peak frequency, whose values at three different spots were almost constant. A difference of about 201 Hz was observed between the peak frequencies of healthy and infected potatoes. The average peak frequencies for chosen spots of tubers with hollow heart were 0.957, 0.942, and 0.918 kHz and 1.157, 1.142, and 1.123 kHz for tubers without hollow heart. A much higher value in the power spectrum of normal tubers was observed than that of hollow tubers. A much higher correlation was observed for the resonant frequencies (R^2^ = 0.97) than for the damping coefficient (R^2^ = 0.65). The built prediction model based on LDA allowed to estimate data with an accuracy of 98% [66]. Applications of ultrasonic, acoustic mechanical methods in quality assessment of potatoes are presented in Table 1.

The conducted research showed the possibility of using ultrasounds to modify the quality parameters by changing their chemical properties of potatoes in the processing industry. Ultrasound at 50 Hz was used to lower the oil content of French fries. It was observed that French fries, before the frying process, subjected to ultrasonic treatment at 28 and 40 kHz and drying at 80 °C for 15 min, showed lower oil absorption (*p* < 0.05) when frying at 150, 170, and 190 °C. It was observed that the use of predrying allowed for a significant reduction in moisture content, which resulted in reduced oil absorption during frying. Ultrasonic waves caused an increase in the vapor pressure around the fried product during frying by moisture migration through microscopic channels and caused reduction of absorption of oil [67]. Kulkarni observed that the oil content of fries that were covered with edible lac resign, plasticized shellac with only 5% increased from 54.24% to 57.87% after being subjected to ultrasound treatments for 10 min at 50 Hz. It was also observed that the application of a 4% concentration of shellac reduces the oil absorption by 44%, and the application of a 4% concentration along with ultrasonic treatment for 5 or 10 min reduces the oil absorption by 46% and 49%, respectively [68]. It was observed that the chips subjected to the drying process, for a mass loss of 80%, 50%, and 20%, respectively, in relation to the mass loss without drying, in combination with the ultrasound treatment before frying, are characterized by a lower fat content, which decreased by 7.42%, 24.10%, and 51.76%. In addition, the chips had more pores and cracks. The hardness of chips also increased with increasing frying time [69].

The effect of ultrasound on chips was also studied. Amaral studied the effect of the use of ultrasound on the quality of vacuum-packed potato chips by sonicating the chips for 5 min at a frequency of 40 kHz in an ultrasonic bath containing 20 g L^−1^ citric acid solution. The treatment maintained the texture and appearance of the chips for up to 12 days at a temperature of 3 ± 1 °C as well as inhibited the growth of microorganisms (Enterobacteriaceae, coliforms, and mesophilic bacteria). However, it did not significantly affect the fat absorption, color, shear strength, or acrylamide content in the chips [70].

Research was conducted on the influence of ultrasound on the properties of the quality parameters of chips: starch properties, water level, oil absorption, and pore characteristics. Tests at a frequency of 20 kHz for 60 min and power of 360 W showed that the initial ultrasonic treatment leads to erosion of the surface of starch granules, and at higher power of 600 W causes disorganization of the starch structure. In addition, changes in bound and immobilized water were observed after using ultrasound pretreatment, which also caused formation of the pores with diameters of 0.4–3 μm and 7–12 μm. At its low power, the content of penetrated surface oil was reduced by 27.31% and the content of structural oil (STO) was reduced by 22.25%, while at high power, the content of surface oil (SO) and PSO was increased by 25.34% and 12.89%, respectively, and STO reduction by 38.05% [71]. It was demonstrated on the example of potato cubes that ultrasounds with a frequency of 35 kHz successfully support and significantly accelerate the freezing process, reducing costs, and at the same time do not cause overcooling of the products. The freezing process has a significant impact on the quality, stability, and sensory properties of chilled products [72]. The influence of pulsating electric field in combination with ultrasounds on frying parameters was investigated. As a result of the treatments, a significant oil reduction of 63.3% and a significant reduction of acrylamide content of 66% were observed. The influence of PEF and ultrasound on the water content was negligible [73]. Applications of ultrasonic mechanical and acoustic methods in quality assessment of processed potatoes are presented in Table 2.

## 5. Application of Artificial Intelligence Methods

Neural networks have been used for many years in agricultural and food industries, for example, for classifying the quality of beans [74], citrus fruits [75], apples [76], plums, tomatoes [77], coffee beans [74,78], and strawberries [79], or detecting plant diseases on plantations [80,81,82,83,84,85,86,87,88,89,90] due to the ease of solving troublesome, difficult, or multidimensional linear and nonlinear problems, which, using traditional software techniques, require the use of complicated algorithms and, thus, a lot of time and dedication.

Teaching a neural network consists of remembering the patterns trained during the learning process and setting the optimal parameters of the network and its hyperparameters using feedforward and backpropagation techniques in such a way as to minimize the prediction error and prepare the network to solve similar tasks on the basis of which the network was taught. The neural network is trained and tested on the basis of a dataset, which is usually divided into two independent subsets: a training subset and a validation subset. The training subset is the largest, covering 75–80% of the entire dataset. It consists of reference data and associated responses. The validation subset is the rest of the dataset: 15–20%. It is necessary to check the accuracy of the network in terms of its ability to generalize the network and thus prevent its overtraining. If the dataset is large enough, a test subset is also extracted from the training set, which is 10–15% of the training set. This is to check the correctness of all network model parameter settings. Individual sets are assessed in terms of classification accuracy for classification models and evaluation of the mean squared error value for regression models [91].

The multilayer feedforward neural networks (MLF-NNs) were used to build a prediction model for classifying external defects based on color segmentation. The results of the research showed a slightly higher efficiency of the MLF-NN model than for the model built using the standard linear discriminant analysis (LDA) technique in combination with the Mahalanobis distance classifier. The results of the prediction accuracy depended on the tested varieties: Bildstar, Bintje, Eigenheimer, Irnene, Sante, and for MLF-NN were 99.2%, 96.9%, 88.1%, 91.6%, and 98.7%, respectively, and for LDA, respectively, were 95.1%, 96.2%, 86.8%, 89.3%, and 98.6% [92].

Research was conducted on the use of neural networks to classify chips based on acrylamide content. The RGB chips model was converted to the HSV model after extracting it using the blue channel. Then, six statistical features distinguishing healthy chips from chips affected by acrylamide were separated: the average value of the red, blue, and green channels, saturation, hue, and brightness, which were used to build the input layer of the neural network model. The set of labeled data consisting of 84 samples was divided in a ratio of 70:30 into a training set and a test set, which allowed after training the network to obtain 99% accuracy of prediction, determined on the basis of the area under the curve method (AUC) [36].

Research was conducted on the use of a neural network to detect defects in Andean potatoes of the Huayro variety caused by pests Epitrix, Gorgojo de los Andes, and other diseases. The paper presents two methods of teaching a neural network and classifying potatoes in terms of defects: learning with a teacher whose training on a set consisting of 4211 27 × 27 pixels of healthy and unhealthy zones allowed for 87.73% accuracy of the prediction model, and adaptive learning by means of which, using 1795 data, the prediction accuracy was 88.2%. It was noticed that the adaptive network uses a smaller set of data needed to train the network than a classic neural network, and its accuracy is slightly higher [93].

Neural networks were used to classify potato varieties Vineta and Denar. The multilayer perception-artificial neural network (MPL ANN) 18:18–51-1:1 neural network model, created on the basis of Statistica software, which was selected from 40 previously determined discriminants (29 parameters defining the color: RGB (red, green, blue); HSV (hue, saturation, value); YCbCr (Y is the luminance component, and CB and CR are the chrominance components of the blue color difference and the red difference); luminance; saturation, which consists of max, min, median, mean, and standard deviation; seven selected aspect ratios, such as Feret coefficient, circularity coefficient (W1, W2), Malinowska coefficient, dimensionless coefficient, LP2 coefficient, Mz4 coefficient geometric features: area, width, height, center of gravity), allowed for the classification of potato varieties with an accuracy of R^2^ = 0.99 [94].

Research was carried out to use neural networks to classify potato varieties Denar, Gala, Nandina, Lord, and Vineta, and to identify damaged potatoes: neural network MLP 102–16-6, built from the input value of 102 descriptors determined using software called “PID system” and MATLAB software, allowed to obtain classification accuracy with the result R^2^ = 0.97, and it was observed that the most important descriptors are standard deviation for red color, standard deviation of the differential component of light intensity and red color in YCbCr, and information about the degree of correlation for the pair minimum value in the GLCM texture [95].

A high-throughput phenotyping method was developed to assess soft rot disease symptoms using Python and MATLAB image analysis software, using neural networks that successfully extract the exact lesions that are in each inoculated tuber. Semantic segmentation based on deep learning was used in the process of disease isolation. The U-net neural network was adapted, which was appropriately modified by adding the normalization and early stop functions. A total of 2700 images were used to train the neural network, which were generated on the basis of 400 previously labeled images using data augmentation techniques such as blurring, rotation, and image sharpening, which were intended to protect the training of the network against overtraining. A high accuracy value of 0.95 was obtained for the identification of diseased areas, which were used for further statistical analysis [96].

Research was carried out on the development of a system for classifying potatoes according to five different sizes and in terms of shape using a 3D camera using neural networks. Two models of neural networks were compared: a classic MLP neural network with one deep layer and one classification layer with the softmax activation function, and a CCN network with three deep layers, one fully connected layer, and one classification layer with the softmax function. It is proven that the neural network is better suited for classification in terms of shape and its accuracy is much higher: 91.6% than the shape prediction accuracy of the MLP network model, which was 67.1%. Both models performed well for size classification, with 94.5% for the CCN network model and 94.4% for the MPL network model. The 3D camera can work stably under LED or fluorescent lighting, because the light source of the 3D camera is infrared radiation. It was noticed that the camera can be successfully used to detect unevenness, damage, and depressions, but it is not sensitive enough to identify surface sprouts [97].

A highly efficient and fast at the conclusion stage (0.417 s/img) poorly supervised teaching method for classifying potato forests into healthy, damaged, green, with a black dot, post-political scab was presented. The method consisted of training the network into six different classes, locating a previously classified defect by CNN based on a defect activation map (DAM), segmenting the classified defect from coarse to fine to obtain information on the size of the defect, and classifying the previously segmented region of damaged or green tubers according to the severity of the defect. The prepared RGB dataset covering 9688 samples was divided and labeled into individual classes. The performance of the neural network models based on the Alex-Net, VGG-16, and GoogLeNet architectures was compared and the average accuracy of the F1-score classification was 0.92, 0.88, and 0.94, respectively, and the classical method with precisely segmented sets and the accuracy of 0.78 was obtained. The lowest accuracy for prediction models Alex-Net, VGG-16, and GoogLeNet was obtained for black dot disease: 0.96, 0.95, and 0.98, respectively. There was a marked improvement in the classification accuracy of the GNet model with the coarse to fine segmentation phase of the classified defect for severely damaged samples 83.6% and slightly green samples 81.7 compared to the model without this phase for severely damaged samples and slightly green samples, 61.8 and 46.2, respectively. F1-score of the entire system was obtained for the combined networks, GoogLeNet, GoogLeNet_Modif of 0.90, 0.88, and 0.89, respectively [98].

Research was conducted on the use of the CNN neural network to classify potato tubers into healthy and five diseases: black dot, black scurf, common scab, and silver scurf. High classification accuracy was obtained for healthy and five diseases: black dot, black scurf, common scab, and silver scurf, respectively, 76.67%, 100%, 100%, 95%, and 97.5%. It was observed that a large dataset is not needed to train a CNN. A total of 20% of the set of training data (493 photos) was enough to train a neural network model whose prediction accuracy was higher than 90% [99].

Research was conducted on the use of visible (Vis) and infrared (NIR) radiation in the range of 350–1100 nm and microwave radiation (SWIR) in the range of 1100–2500 nm to detect internal defects in washed potatoes such as brown rot, empty heart, and black heart. For both examined radiation ranges, the most important wavelengths were determined for the VisNIR and SWIR models, respectively, 861, 883, and 998 nm and 1539, 1858, and 1896 nm, using a hybrid model consisting of artificial neural networks (ANN) and a cultural algorithm (CA). The prediction model was built on the basis of the prediction agreement of at least three of the four classification models: hybrid competitive ANN and imperialism algorithm (ANN-ICA), ANN and harmony search algorithm (ANN-HS), k-nearest neighbors analysis (KNN), and linear discrimination analysis (LDA). A slight difference was observed between the accuracy of the model using the three wavelengths and all spectral data, which for VisNIR and SWIR were 83.4, 86.1 and 94.1, 96.3, respectively [100]. Applications of artificial intelligence methods in quality assessment of potatoes are presented in Table 3.

## 6. Application of Imaging Techniques for the Qualitive Evaluation of Potatoes

Computer vision systems have been used in the food industry for many years to control food quality, e.g., vegetables [101,102] and fruits [103,104], as well as meat [105,106] and fish [107,108], due to numerous advantages. Computer vision systems enable noninvasive, noncontact, hygienic, rapid, high recognition accuracy, repeatable assessment of product quality. However, the recognition accuracy is subject to error under inappropriate lighting, high humidity, and high noise conditions [109]. They enable fully automated cost-effective quality assessment systems that can replace manual visual inspection and hence eliminate errors and inconsistencies in results [110]. In Poland, optoelectronic sorters, due to their uncompetitive prices in relation to mesh sorters, are gradually gaining popularity. Optical sorters also allow you to sort the product in terms of color, impurities, and damage [49].

Optoelectronic sorters consist of a conveyor, a vision system, and a mechanical separator. The vision system, which includes a camera, takes a picture of the product and, based on the analysis of the image by an industrial computer, classifies it in real time in terms of size, defects, external damage, and contamination. It transfers product information to the controller that controls the mechanical separator. Unlike previous solutions, the handling machine only needs one person to supervise the system. Thanks to this, the machines allow fractionation of vegetables into a larger number of groups [49].

### Imaging in Visible Light

An automatic control station equipped with a CCD camera was developed for the machine classification of potatoes in terms of size and shape. Potato diameter (size) predictions were estimated based on calculation of the Euclidean distance between the vertex points determined using the boundary tracing algorithm, thanks to which the diameter prediction error was less than 1.5%. The shape was analyzed using Fourier descriptors (FDs). The accuracy of the vision system classification of stationary tests was 97–98%, while for dynamic tests whose classification efficiency was 180/h, due to the unfavorable impact of potato movement on the shape analysis algorithm, the accuracy was 77–88% [111].

A PC-based vision system was developed for real-time evaluation of potato quality in terms of potato weight, cross-sectional diameter, shape, and color. The implemented vision system equipped with a CCD camera is able to classify 50 potatoes per second or 16 potatoes per second, working in the mode of increased accuracy, in which the classification is carried out on the basis of photos from different angles. The shape classification was estimated based on the ellipse descriptor. Greens were detected using the thresholding method, based on the hue value in the hue–saturation–value (HSV) space. The cross-sectional diameter was determined as a byproduct of shape inspection (minor axis of the ellipse fit). The weight was calculated based on the projected area of the potato tuber for the viewing angle of the camera. The average efficiency of weight and diameter estimation was 91.2% and 88.7%, respectively. The prediction accuracy of the shape and color models was 85.5% and 78%. The overall classification accuracy of the sorter was 86.5% [112].

A high-speed potato grading machine was developed for quality control in terms of size, shape, external defects such as greening, mechanical damage, and pathogens: rhizoctonia, silver scab, common scab, cracks, and growth cracks. The vision system of the sorter was equipped with a three CCD line camera and a set of mirrors with the help of which, during the flight of the potato under the camera, a 360-degree view of the tuber is created, on the basis of which the system performs the image processing and classification tasks. The sorter with a belt speed of 1.5 m/s has a capacity of 12 tons/h or about 50 potatoes/s. The majority of external defects and diseases were identified by color segmentation that used LDA linear discriminant analysis, the Mahalanobis distance classifier, and features such as area, eccentricity, and central moments that helped in differentiation between similar colored defects. In order to detect misshapes, the Fourier-based shape transform was applied to the contours of the tuber. Shape prediction accuracy was 89.2% [92].

Comparative research was conducted in order to find the simplest, most effective, and least computationally complex method for identifying the size and shape of a potato with the usage of vision system. Two methods using a minimal rectangle and an ellipse R ratio were analyzed. Studies have shown that in the case of a constant orientation of the sample, the rectangular method works better, and its error is <4%, while in the case of redundant sample orientation, the method using an ellipse is better and its error is <7%. The created prediction model based on the eclipse method allowed for differentiation of potatoes of different varieties, Atlantic, Xiang No. 1, and Favotita, into round, long cylindrical, and oval shapes, with the accuracy level of 98.8% [113].

Yondsheng developed a high-performance digital-image-based method for estimating the L/W ratio on tubers of red, white, and rust varieties. An image processing algorithm was developed which consisted of an image segmentation by thresholding the gray image obtained by the difference in the intensity of the red and green band pixels and its subsequent binarization using the Otsu method. The application of a marker-controlled watershed segmentation algorithm was used to divide tubers that were attached to each other. L/W ratios were calculated based on the minimum rectangles created for each potato. High accuracy of L/W ratio estimation was achieved for all varieties. The average accuracy for the Russet and white variety was 96%, while for the red variety it was 84–100%, of which 50% of the tubers were underestimated or overestimated due to the moderate correlation coefficient *p* = 0.5688. For domain Russet and white potatoes, the correlation coefficient was 0.94 and 0.97, respectively [114].

A vision system equipped with a CCD camera was developed, which estimated the length-to-width ratio of fresh potato tubers of various varieties (red, yellow, rust) and field varieties Alturas and Bondi on an integrated high-performance conveyor belt with a high speed of moving cup-type trays in which tubers were sinuated and transferred during process of 43 trays/min, 62 trays/min, and 86 trays/min that corresponded to 86, 129, and 172 rpm of speed of conveyor, respectively. The developed algorithm extracted two potato tubers from the defined ROI region in the image and determined the minimum rectangular area of the tuber on the basis of which its length and height were estimated. A high measurement accuracy of 95% was obtained for fresh tubers, as was a high value of the correlation coefficient between the estimated measurement from the photos and the measurement using a caliper (*p* < 0.01), and 76–100% accuracy for field varieties. It was observed that the accuracy of the classification is not affected by the quality of the measurement [115].

Research was conducted on the use of vision systems to identify external defects such as green, germination, and lesion of potatoes in the visible environment. In order to segment the potato image and eliminate the background color, the R-B<T1 and G-B<T2 thresholding method were used, then the RGB color model was transformed into the HSV model, the S values were grayed out, and the image was binarized. In order to identify the green skin of the potato tuber, we obtained information from three color space models: RGB, HSV, LAB, from which, based on the statistical analysis of healthy and diseased skin, independent threshold parameters were extracted: R–G from the RBG model, H threshold parameters from the HSV model, and parameter A from the LAB model. It was observed that the Euclidean method is susceptible to the influence of light, and the color threshold method is also affected to hue aberration. A procedure for germination and damage identification based on the gray variance of the Laplace operator was proposed [116].

Research was conducted on the use of an RGB-D camera to measure the volume of a potato and classify it on the basis of its shape into regular/irregular. The RGB-d camera was equipped with a depth measurement function based on stereoscopic vision. A total of 120 tubers were allocated for the test harvest, which were experimentally divided evenly into two groups: 60 tubers with the most regular shape and 60 tubers with an irregular shape. An algorithm was created that calculated the volume of the potato on the basis of depth images taken on both sides of the potato and allowed the classification of tubers in terms of regular/irregular shape with a relatively high prediction error of 9% for the regular shape and 30% error for the irregular shape, resulting from the loss of information about depth at the contours of the tuber [117].

Stationary research was conducted on the development of a vision system for identifying the internal disease of potato tubers black heart based on light transmission imaging technology using a monochromatic CCD camera and LED lighting with a radiation frequency of 700 nm. It has been observed that black heart disease weakens the emitted signal, forming black spots on the image. The gray trend of the healthy potato was uniform, while the gray value of the diseased potato was low. On the basis of 30 samples, a calibration model was created for a healthy potato based on the average shade of gray of the tuber pixels and the diameter of the potato. A high correlation was observed between the diameter of the potato and the average shade of gray for a healthy potato, the correlation coefficient of which was R^2^ = 0.83. The accuracy of the model prediction on the validation set was 75% [118].

Similar research on the vision system for identifying internal disease of potato tubers black heart based on light transmission imaging technology was carried out using CCD and LED lighting with a radiation frequency of 705 nm operating online with an integrated conveyor belt and sorting unit. Based on the analysis of the transmission image, of its 90% diameter of the potato tuber, on the basis of the correlation of six parameters, average grayscale value of the entire potato image area (Aw), high (Ah) and low (Al) grayscale area, standard deviation of the entire potato image area (Sw), and low (Sl) and high (Sw) grayscale area, a prediction model was created using multiple linear regression (MLR), which selected the most important parameters: Aw, Sw, Sl, and Sh. The accuracy of the prediction model on the training set was 94.4%, while on the validation set it was 88.9% [119].

The effectiveness of the use of a camera with depth technology for the classification of washed potatoes has been successfully proven. This allows not only to measure the length, width, and thickness of the vegetable, but also to using developed image processing algorithms such as grid calculation (GS) and the oval difference degree (ODD), which enable the recognition of external defects with a high accuracy of up to 88%, such as dimples, bulges, or irregular shape, and the prediction of potato weight with an accuracy of 90% (7.7 g) thanks to the developed model based on a volume-based linear regression algorithm. A high correlation of 0.99 was observed between potato volume and its weight for tubers of the same species. The time for the classification of one potato was rather long: 140 ms. The 3D image is effective in detecting bumps and hollows located in the center of the tuber, which are clearly visible through the shade of gray pixels. In a 2D color image, such deformations are difficult to detect due to too-small difference in pixel color between the healthy and deformed part of the tuber [120].

Research was carried out on the development of a computer vision system integrated with a roller conveyor, with high efficiency, for grading potatoes in real time on the basis of regularity of shape and size. On the basis of the linear discriminant analysis, the most important parameters characterizing the regularity of potatoes were selected: two shape features: roundness and extent, and four features of the Fourier-shape-based descriptors: S1, S2, S3, and S4. These were used to build a predictive model, as a result of which, for the training dataset, 100% accuracy of prediction of round potatoes was obtained, and 79% accuracy of detection of misshapen potatoes. The average performance of the classification model was 96.5%. For the model tested in real-time in-line operation at a speed of 1 m/s, the accuracies for regular and misshapen potatoes were 75% and 98.8%, respectively. The overall accuracy of the predictive model was 96.2%. The vision system offered 100% accuracy for the weight size of small: <40 cm^2^, medium: 40–50 cm^2^, large: 55–70 cm^2^, and very large: >70 cm^2^ potatoes [121].

Application of vision systems in light range methods in quality assessment of potatoes are presented in Table 4.

## 7. Conclusions

A mechanical, and the new noncontact, technology presented in the article allows to replace conventional sorting methods and can provide a lot of valuable information on the chemical and physical parameters of potato tubers. The correct classification of potatoes on the basis of internal crop defects is extremely important for storage. Early detection of internal diseases of tubers will limit the spread of pathogens of infected potatoes, which will reduce storage losses. In the case of potatoes intended for seed material, correct classification based on internal defects will reduce the occurrence of diseases on plantations, which will affect the quality of crops and their size. In the face of the increased demand for high-quality products resulting from the globalization of the market and ensuring the safety of food quality, there is a growing demand for precise sorting machines for classifying agricultural products. This article is limited to mechanical sorters, ultrasonic methods, and vision systems based on image processing algorithms and artificial intelligence to assess the quality of chemical and physical parameters. Mechanical sorters allow one to grade potatoes by length, width, or thickness. Ultrasonic techniques are used to analyze physicochemical parameters and internal defects as well as to modify physicochemical quality parameters during food processing. In order to detect potato hollow heart disease, research is being carried out on the use of ultrasonic and acoustic methods. Ultrasonic methods have also been used to improve the quality of chips and fries by reducing the content of fat and acrylamide and accelerating the freezing process. Vision systems based on image analysis algorithms enable the most complete classification of potatoes with high accuracy due to size, shape, external-greening, mechanical correctness, and diseases. The accuracy of the estimation can be increased by using more advanced image processing algorithms. Vision systems based on neural networks allow the development of even more demanding and complex potato grading applications while maintaining high accuracy. They were used to identify external defects caused by pathogens or mechanical damage, size, shape, differentiation of potato varieties, and also to estimate the content of acrylamide in chips. Further research on ultrasonic methods and vision systems based on image processing algorithms or artificial intelligence will certainly increase the accuracy of estimating chemical and physical parameters and thus contribute to maintaining the appropriate food quality requirements set out in legal regulations and ensure health safety for consumers of products introduced to the market. The development of new technologies will increase the accuracy of the classification of potatoes of different varieties. The most promising of the methods described above are techniques using artificial intelligence, and the most dynamic development is observed in this direction.

## Figures and Tables

**Table 1:** Applications of ultrasonic, acoustic mechanical methods in quality assessment of potatoes.

Quality Attribute	Specimen	Condition of Measurements	Method	Frequency [Hz]	Power [W]	Discriminant	P [%]	Ref.
Size	Tubers	Online	Mechanical	-		-	95	[52]
Density	Tubers	Underwater	Ultrasound	-		Power spectral moment.	-	[65]
Hollow heart	Tubers	Stationary	Acoustic impact	-		Integrated absolute value of microphone output signal, number of data points in the digitized microphone signal following impact.	98	[66]
Hollow heart	Slice	Underwater	Ultrasound	175		-	-	[60]
Hollow heart	Tubers	Underwater	Ultrasound	100,000		Attenuation coefficient.	92	[61]
Hollow heart	Tubers	Dry-coupling	Ultrasound	250,000		Power spectral moment.	-	[62,63]
Hollow heart	Tubers	Dry-coupling	Ultrasound	50,000	0.22	Transmission loss.	98	[64]

**Table 2:** Application of ultrasonic methods in quality assessment of processed potatoes.

Quality Attribute	Application	Method	Frequency [Hz]	Time [s]	Power [W] Quality Attribute	Ref.
Better freezing	Cubes	Ultrasound	35,000	8	300	[72]
Oil uptake (*p* < 0.05) Moisture content (*p* < 0.05)	Fries	Ultrasound	20,000 40,000	900	160	[67]
Fat content reduction by 63.3% Acrylamide reduction by 66%	Chips	Ultrasound	-	180	1000	[73]
PSO increase by 12.89% STO reduction by 38.05% TO reduction by 13.37% SO increase by 25.34%%	Chips	Low ultrasound	20,000	3600	600	[71]
PSO reduction by 27.31% STO reduction by 22.25% TO reduction by 23.92%	Chips	Low ultrasound	20,000	3600	360	[71]
Reduction of oil content by 7.42% Reduction of oil content by 24.10% Reduction of oil content by 51.76%	Chips	Ultrasound	-	7200	360	[69]
Fat content reduce by 3%	Fries	Ultrasound	50	600	-	[68]
Maintained the quality attributes of chips	Chips	Ultrasonic	40,000	300	200	[70]

**Table 3:** Applications of artificial intelligence methods in quality assessment of potatoes.

Method	Measurement	Prediction	Breed	Number of Samples	Discriminant	Accuracy	Ref.
Camera RGB	Stationary, slices, supervised learning	Acrylamide	-	84	Average value of the red, blue, green channels, saturation, hue, and brightness.	0.99	[94]
Camera RGB	Stationary, tubers, supervised learning	Defects	Huayro	4211	A set of 27 × 27 pixels of healthy and unhealthy zones.	0.88	[93]
Camera RGB	Stationary, tubers, adaptive learning	Defects	Huayro	1795	A set of 27 × 27 pixels of healthy and unhealthy zones.	0.88	[93]
Camera RGB	Stationary, tubers, stationary, tubers,	Varieties	Vineta, Denar	2000	RGB, HSV, YCbCr, luminance, saturation, (max, min, median, mean, standard deviation), Feret coefficient, circularity coefficient (W1, W2), Malinowska coefficient, dimensionless coefficient, LP2 coefficient, Mz4, area, width, height, center of gravity.	0.99	[94]
Camera RGB	Stationary, tubers, stationary,	Varieties, defected	Denar, Gala, Nandina, Lord, Vineta	21,000	Hue space models, aspect ratio, and image texture.	0.97	[95]
Camera RGB	RGB images	Disease symptoms to bacterial soft rot		2700	RGB images.	0.95	[96]
Depth Camera,	Stationary, tubers, MLP	Size		5691	Depth images.	0.944	[97]
Depth Camera,	Stationary, tubers, CNN	Size, shape		5691	Depth images.	0.945	[97]
Camera RGB	Stationary, tubers	Healthy, black dot, black scurf, common scab, silver scurf		2465	A set of images of unhealthy zones in grayscale 224 × 224 pixels.	0.77, 1.00, 1.00, 0.95, 0.97	[99]
Camera RGB	Stationary, Tubers, GoogLeNet+OC-SVMSeg+SVMGravity (GNet)	Healthy, light damaged, serious damaged, light greening, serious greening, black dot, common scab, black scurf	Agata, Monalisa, Gourmandine, Annabelle, Caesar, Charlotte and Marilyn	9688	RGB images	0.88	[98]
Camera RGB	Stationary, Tubers, GoogLeNet_Modif + OC-SVMSeg + SVMGravity (GNet_Modif)	Healthy, light damaged, serious damaged, light greening, serious greening, black dot, common scab, black scur	Agata, Monalisa, Gourmandine, Annabelle, Caesar, Charlotte and Marilyn	9688	RGB images	0.89	[98]
Camera RGB	Stationary, Tubers, Combination of (GNet and GNet_Modif)	Healthy, light damaged, serious damaged, light greening, serious greening, black dot, common scab, black scurf	Agata, Monalisa, Gourmandine, Annabelle, Caesar, Charlotte and Marilyn	9688	RGB images	0.90	[98]

**Table 4:** Application of vision systems in light range methods in quality assessment of potatoes.

Method	Measurement	Prediction	Breed	Number of Samples	Discriminant	Accuracy Training Set [%]	Accuracy Test Set [%]	Error [%]	Ref.
CCD	Stationary	Shape, size	-	65	Euclidean distance, Fourier descriptors (FDs).		77–88		[111]
CCD	Online	Shape, size	-	65	Euclidean distance, Fourier descriptors (FDs).		97–98		[111]
CCD	Online	Weight, cross-sectional diameter, shape, color. Average:	3	200	Projected area of the potato, ellipse (minor axis), ellipse, hue–saturation–value (HSV).		91.2, 88.7 85.5 78.0 86.5		[112]
CCD	Stationary	Round Oval Long cylinder	Atlantic Xiang No.1 Favotita	228	Ellipse.		98.8		[113]
Depth 3D	Stationary	Regular shape	random	60	Volume measurement based on depth image processing algorithm.			9	[117]
Depth 3D	Stationary	Non-regular shape	random	60	Volume measurement based on depth image processing algorithm.			30	[117]
CCD	Tubers, inline	Shape: regular, misshapen	-	410	Shape prices: roundness, span, and Fourier shape size characteristics: S1, S2, S3, S4.	100 79	98.8 75		[121]
CCD	Tubers, inline	Size	-	410	Shape prices: roundness, span, and Fourier shape size characteristics: S1, S2, S3, S4.		100		[121]
Depth 3D	Stationary	Size	Jizhangshu No. 8	60	Minimal bounding rectangle.				[120]
Depth 3D	Stationary	Mass	Jizhangshu No. 8	100	Based on volume prediction.		90		[120]
Depth 3D	Stationary	Bump Hollow Surface area shape	Jizhangshu No. 8	97	Grid calculation (GS), the oval difference degree (ODD).		100		[120]
CCD+LED 700 nm	Stationery	Black heart	Netherlands	38	Average grayscale value of the entire potato image area (Aw), standard deviation of the entire potato image area (Sw), low (Sl) and high (Sw) grayscale area.	83	75		[118]
CCD	Online	L/W	Russet Red Yellow Alturas Bondi	16 13 14 25 25	Minimal bounding rectangle	93.3–97.2 95.7–100 95.6–98.8 76.8–99.6 76–81.2			[119]
CCD+LED 705 nm	Online	Black heart	Netherlands	72	Minimal bounding rectangle	94.4	88.9		[115]
Camera	Stationary	L/W	White Russet Red	19	Minimal bounding rectangle	Avg 96 Avg 96 84–100			[114]
CCD	Stationary	Misshapen	various	130	30 Fourier descriptors		100		[92]
CCD	Stationary	Misshapen	various	130	20 Fourier descriptors		98.8		[92]
CCD	Stationary	Misshapen	various	130	10 Fourier descriptors		97.6		[92]
CCD	Stationary	Cracks	various	95	Color segmentation, eccentricity, area		100		[92]
CCD	Stationary	Cracks	Bintje	64	Color segmentation, eccentricity, area		96.8		[92]
CCD	Stationary	Greening, common scab, silver scab Rhizoctonia	Bildstar Bintje Eigenheimer Irene Sante	-	Color classification		95.1 96.2 86.8 89.3 98.6		[92]

## Data Availability

Data are available by contacting the authors.
